# Immunonutrition with Omega-3 Fatty Acid Supplementation in Severe TBI: Retrospective Analysis of Patient Characteristics and Outcomes

**DOI:** 10.1089/neur.2024.0005

**Published:** 2024-06-19

**Authors:** Roy A. Poblete, Jesus Pena, Grace Kuo, Fawaz Tarzi, Peggy L. Nguyen, Steven Y. Cen, Shelby Yaceczko, Stan G. Louie, Meghan R. Lewis, Matthew Martin, Arun P. Amar, Nerses Sanossian, Gene Sung, Patrick D. Lyden

**Affiliations:** ^1^ Department of Neurology, University of Southern California Keck School of Medicine, Los Angeles, California, USA.; ^2^ Department of Radiology, University of Southern California Keck School of Medicine, Los Angeles, California, USA.; ^3^ UCLA Health, University of California, Los Angeles, California, USA.; ^4^ Department of Clinical Pharmacy, University of Southern California School of Pharmacy, Los Angeles, California, USA.; ^5^ Department of Surgery, University of Southern California Keck School of Medicine, Los Angeles, California, USA.; ^6^ Department of Neurological Surgery, University of Southern California Keck School of Medicine, Los Angeles, California, USA.; ^7^ Zilkha Neurogenetic Institute, University of Southern California, Los Angeles, California, USA.

**Keywords:** head trauma, inflammation, nutrition, traumatic brain injury

## Abstract

Early evidence-based medical interventions to improve patient outcomes after traumatic brain injury (TBI) are lacking. In patients admitted to the ICU after TBI, optimization of nutrition is an emerging field of interest. Specialized enteral nutrition (EN) formulas that include immunonutrition containing omega-3 polyunsaturated fatty acids (n-3 PUFAs) have been developed and are used for their proposed anti-inflammatory and proimmune properties; however, their use has not been rigorously studied in human TBI populations. A single-center, retrospective, descriptive observational study was conducted at the LAC + USC Medical Center. Patients with severe TBI (sTBI, Glasgow Coma Scale score ≤ 8) who remained in the ICU for ≥2 weeks and received EN were identified between 2017 and 2022 using the institutional trauma registry. Those who received immunonutrition formulas containing n-3 PUFAs were compared with those who received standard, polymeric EN with regard to baseline characteristics, clinical markers of inflammation and immune function, and short-term clinical outcomes. A total of 151 patients with sTBI were analyzed. Those who received immunonutrition with n-3 PUFA supplementation were more likely to be male, younger, Hispanic/Latinx, and have polytrauma needing non-central nervous system surgery. No differences in clinical markers of inflammation or infection rate were found. In multivariate regression analysis, immunonutrition was associated with reduced hospital length of stay (LOS). ICU LOS was also reduced in the subgroup of patients with polytrauma and TBI. This study identifies important differences in patient characteristics and outcomes associated with the EN formula prescribed. Study results can directly inform a prospective pragmatic study of immunonutrition with n-3 PUFA supplementation aimed to confirm the biomechanistic and clinical benefits of the intervention.

## Introduction

Traumatic brain injury (TBI) is a significant cause of morbidity and mortality that affects all populations, regardless of gender, age, ethnicity, race, and socioeconomic background. Severe TBI (sTBI) results in >60,000 deaths in the United States (US) annually and over 5 million persons living with TBI-associated disability.^1,2^ First-year health care costs are estimated to be $2 billion in survivors.^2^ Despite the large health and socioeconomic impact, there are still no Level 1 recommendations for medical interventions to improve outcomes in moderate and sTBI.^3^ The pathophysiology of sTBI is complex, with several early proinflammatory molecular and cellular changes occurring in the immediate and delayed phases after trauma.^4^ Although these pathways are thought to be largely beneficial to support initial reparative processes, they may also contribute to adverse clinical symptomatology, metabolic stress, and disease progression if left untreated. In this context, early in-hospital interventions targeting inflammation and metabolism may prevent secondary brain injury and systemic complications that contribute to poor clinical outcomes after TBI.

Optimization of nutritional status with early (within 48–72 h of admission) enteral nutrition (EN) is an emerging therapeutic strategy in critically ill and trauma populations.^5,6^ The goal is to both prevent malnutrition and provide essential nutrients to promote cellular and neurological recovery. After sTBI, malnutrition occurs in up to 76% of intensive care unit (ICU) patients and is associated with increased mortality and poor functional outcomes.^7–9^ Its high incidence is multifactorial, with important contributions from dysphagia, increased metabolic demands, altered digestive and metabolic function, and variable feeding practices while in the ICU. Altogether, critical illness and malnutrition in TBI result in a proinflammatory, immunosuppressed and catabolic state that predisposes individuals to complications such as fever and nosocomial infection, which are both independent predictors of poor outcome.^10,11^ Early nutritional support with specialized dietary formulas that include immunonutrition with omega-3 polyunsaturated fatty acid (n-3 PUFA) and amino acid supplementation is a novel approach to optimize nutrition and promote recovery after TBI; however, their clinical effect has not been rigorously studied in acute neurological disease. In pre-clinical models, administration of inflammation-resolving bioactive lipids, including the n-3 PUFAs eicosapentaenoic acid (EPA) and docosahexaenoic acid (DHA), has robust evidence for neuroprotection, anti-inflammatory properties, and improved cellular energetics.^12^

The purpose of this descriptive retrospective observational study was to compare the population characteristics of sTBI patients who received EN immunonutrition with n-3 PUFA supplementation versus those who received standard, polymeric EN, which did not contain the added components of immunonutrition. The supplementation of n-3 PUFAs in TBI is an appealing therapeutic strategy given their proposed anti-inflammatory effect, widespread availability, safety profile, relative low cost, and general feasibility in implementing into ICU standards of care. When incorporated into universal strategies to optimize nutritional status, such novel approaches might lead to meaningful impact on patient and hospital outcomes. The results of this study are anticipated to inform future prospective clinical trials investigating the clinical effect of immunonutrition, including n-3 PUFA supplementation in moderate-to-severe TBI and other acute neurological diseases.

## Methods

### Study population

This is a single-center retrospective observational study of sTBI conducted in Los Angeles, California. The single study site, Los Angeles General Medical Center (formerly named LAC + USC Medical Center at the time of patient admission) is a high-volume Level 1 trauma center and the largest safety net hospital in the Western United States. This descriptive study was considered hypothesis-generating, aimed to characterize differences in patient populations based on interventions provided as clinically indicated, with no primary hypothesis testing.

The target population included patients with sTBI who received EN in the ICU. Study subjects were identified from the institutional trauma registry that is maintained by the Division of Trauma and Acute Care Surgery. A 5-year period between July 2017 and July 2022 was studied. Sample study subjects met the following inclusion criteria: age ≥ 18 years, diagnosis of sTBI (history and radiographical findings consistent with TBI, Glasgow Coma Scale score [GCS] ≤ 8), ≥14-day ICU length-of-stay (LOS), prescribed and received EN during the majority (>7days) of the 14-day ICU period. No time cutoff for initiating EN was used. Patients who presented with GCS = 3 with dilated pupils, or those who did not tolerate planned EN initiation due to gastric dysmotility were excluded from the analysis. These inclusion and exclusion criteria were chosen to ensure that study subjects were sufficiently exposed to the intervention of interest. The study population included both isolated head trauma (IHT) and polytrauma with TBI. It was preplanned to study subgroups of IHT and polytrauma to determine how these populations might differ in their baseline characteristics and response to EN.

### Intervention

The intervention of interest was EN using an immunonutrition formula containing n-3 PUFAs from fish oil. Those who received EN with immunonutrition, either through gastric or postpyloric feeding tube, were compared with those who received standard polymeric EN formulas not classified as immunonutrition. Immunontrition formulas have been developed for their proposed anti-inflammatory properties, to promote immune system function, and support biosynthesis and protein needs in high-risk critically ill and trauma patients.^13^ At the study site, EN products are supplied by Abbott Nutrition^®^. Peptide-based products classified as immunonutrition that were prescribed to study subjects were Vital High Protein^TM^ (containing EPA = 2.3 g/L, DHA = 0.9 g/L, vitamin D, C, and E), Vital AF 1.2 Cal^TM^ (containing EPA = 2.7 g/L, DHA = 1.1 g/L, vitamin D, C, and E) and Pivot^TM^ (containing EPA = 2.6 g/L, DHA = 1.1 g/L, arginine, glutamine, vitamin C and E). In addition to n-3 PUFAs, formulations also contain essential vitamins and amino acids as listed to support immunomodulation, antioxidant activity, and cellular peptide biosynthesis. Given the retrospective, observational nature of this study, the choice of specific EN formulation used, time to initiation, dose, and duration were determined by clinical judgment. At the study site, a clinical registered dietitian is involved in the nutritional management of ICU patients who require EN, including determining the formulation prescribed, goal dose rate to meet caloric needs, and reinitiation schedule after pauses in EN delivery. Although not protocolized at our institution, immunonutrition formulas are often chosen for patients with high protein requirements because of critical illness or wound healing as described.

### Data collection and study variables

The institutional trauma registry was queried to identify patients meeting inclusion criteria. The sample population consisted of remaining subjects after inclusion and exclusion criteria were applied. Data that were not contained in the trauma registry was retrospectively collected from the electronic medical record (EMR) by study personnel. Demographic and baseline characteristics, including comorbidities were obtained, as well as the EN product used and time to initiation. For outcome measures, clinical markers of inflammation were recorded, including peak 14-day values of the common acute phase reactants, C-reactive protein (CRP, mg/L) and white blood cell count (WBC, ×10^9^/L), and measures of fever occurrence. Both the number of calendar days with fever (any occurrence of temperature >37.8°C) and cumulative fever burden (arithmetic sum of daily peak fever >37 over the 14-day study period) were collected. This measure of fever burden has been validated as an independent predictor of outcomes after TBI.^10^ Patient outcomes, including incidence of culture-positive infection during the 14-day ICU period, ICU and hospital LOS, early GCS recovery (post-TBI day #7 GCS), and discharge status were analyzed. The outcome measures were chosen based on data that could be feasibly collected through retrospective EMR review. This study was designated IRB exempt by the University of Southern California Institutional Review Board.

### Statistical analysis

Data normality for continuous variables was assessed by histogram and Shapiro–Wilk test. Univariate analyses for baseline differences by study groups were conducted by Chi-square test for categorical variables and independent *t-test* or Wilcoxon rank-sum test for continuous variables, depending on data normality. The comparison of inpatient mortality rate was conducted using multivariate Poisson regression with log link function and adjusted for confounders, which were defined as baseline variables with large differences between groups that were clinically important but not necessarily reaching a *p* < 0.05 threshold. There were three variables that met the definition of confounder to the intervention effect: age, gender, and occurrence of noncentral nervous system (non-CNS) acute surgery. Using the log link function for Poisson modeling, the reported associations were interpreted as relative risks (RRs). The overdispersion assumption for Poisson regression was investigated through the goodness-of-fit test using Pearson Chi-square/DF ratio. When this ratio was larger than 1, a negative binomial distribution was used. Differences in hospital and ICU LOS were tested by multivariate generalized linear models and adjusted for potential confounders as described above. The interaction between IHT and polytrauma subgroups and intervention groups was tested in both models. A stratified intervention effect by IHT was illustrated for trend of effect modification. Missing data for laboratory values were excluded from the analysis. SAS 9.4 was used for all data analyses.

## Results

**FIG. 1.**
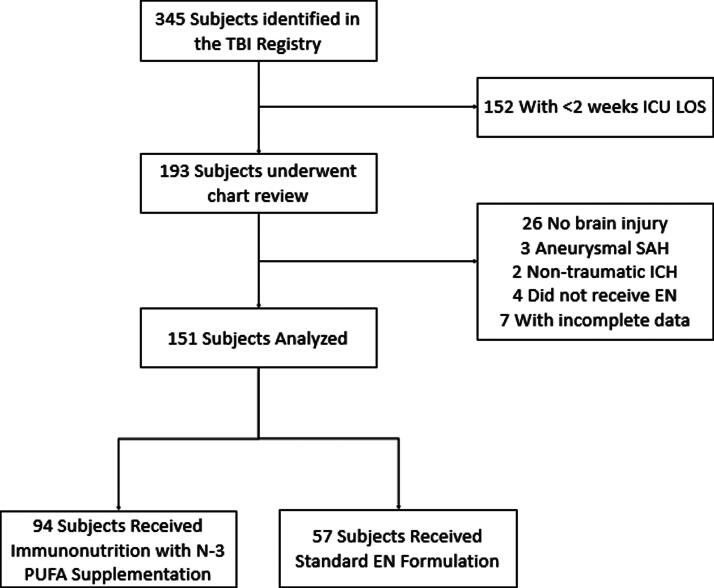
Flow Chart of the Study Population. TBI: traumatic brain injury; ICU LOS: intensive care unit length of stay; SAH: subarachnoid hemorrhage; ICH: intracerebral hemorrhage; EN: enteral nutrition; N-3 PUFAs: omega-3 polyunsaturated fatty acids.

A total of 151 patients with sTBI were analyzed (Fig. 1), of which 61 patients had isolated TBI and 90 patients had polytrauma with associated TBI. Baseline characteristics are shown in Table 1. In the study population, the average age was 43 years, 85% were male and 60% were classified as Latinx/Hispanic. Ninety-four patients (62.3%) received EN with immunonutrition containing n-3 PUFA supplementation and 57 (37.7%) patients received standard, polymeric EN formulations. Compared with the standard EN group, the treatment group who received immunonutrition with n-3 PUFA supplementation were slightly younger (41.2 vs. 46.5, *p* = 0.07) and were more likely to be male and Latinx/Hispanic. The immunonutrition group more commonly sustained motor vehicle accident as the mechanism of injury and were significantly more likely to have polytrauma and require emergent non-CNS surgery. There were no statistically significant differences in baseline comorbidities between the primary study groups and initial GCS was nearly identical. Vital AF^TM^ was the most prescribed immunonutrition formula and was used in 47.9% of patients in the treatment group. Baseline weights and time-to-initiation of EN between study groups was similar (49.6 h in the immunonutrition group vs. 52.8 h in the standard group).

**Table 1.** Characteristics of the Study Subjects at Baseline

Variable	Total (*n* = 151)	Immunonutrition EN group (*n* = 94)	Standard EN group (*n* = 57)	*p* value
Age (mean)	43.2	41.2	46.5	0.07
Male gender (%)	81.6	86.5	73.1	0.05
Hispanic ethnicity	59.6	64.9	50.9	0.23
Comorbidities				
Prior CVA/TBI	2.7	2.1	3.5	0.61
DM	4.6	2.1	8.8	0.06
CAD	0.7	1.1	0.0	0.43
CKD	0.0	0.0	0.0	0.43
TBI mechanism				0.17
Fall	22.5	16.0	33.3	
MVA	32.5	38.3	22.8	
Assault	9.3	8.5	10.5	
Auto vs. Ped	26.5	27.7	24.6	
TBI subtypes				
Contusion	65.6	68.1	61.4	0.4
SDH	79.5	75.5	86.0	0.12
EDH	13.3	13.8	12.3	0.79
SAH	82.8	84.0	80.7	0.6
Skull fracture	53.6	54.3	52.6	0.85
Isolated head trauma	40.4	34.0	50.9	0.04
Non-CNS surgery	11.9	16.0	5.3	0.05
Admission GCS	4.8	4.7	5	0.39
Baseline Wt (kg)	78.5	79.4	77.0	0.58
Hours to EN	50.8	49.6	52.8	0.57
Feeding product				<0.01
Vital AF	29.8	47.9	0.0	
Vital HP	14.6	23.4	0.0	
Pivot	17.9	28.7	0.0	
Other	37.8	0.0	0.0	

Results are displayed as means for continuous variables and % for categorical variables.

CVA/TBI, cerebrovascular accident or traumatic brain injury; DM, diabetes mellitus; CAD, coronary artery disease; CKD, chronic renal disease; MVA, motor vehicle accident; Ped, pedestrian; SDH, subdural hemorrhage; EDH, epidural hemorrhage; SAH, subarachnoid hemorrhage, CNS, central nervous system; GCS, Glasgow Coma Scale score; Wt, weight; EN, enteral nutrition; Vital AF, Vital Advance Formula^TM^; Vital HP, Vital High Protein^TM^; Pivot^TM^.

In unadjusted univariate analysis, peak 14-day ICU WBC and CRP values were similar between study groups (Table 2). Both 14-day cumulative fever burden (15.1 vs. 15.2, *p* = 0.75) and calendar days with fever (8.4 vs. 8.6, *p* = 0.74) were no different in those prescribed immunonutrition versus standard EN formulations. The observed culture-positive infection rate was lower in the immunonutrition group compared with the standard group, but this did not reach statistical significance (80.9% vs. 86.0%, *p* = 0.42). Individually, there was no significant difference in rates of cerebrospinal fluid, respiratory, blood, urine, or wound infections. The presence of ileus on ICU day 14 was similarly rare in both groups, and the number of calendar days with nothing-by-mouth (NPO) status was nearly identical (3.7 days with immunonutrition vs. 4.0 with standard EN). Those receiving immunonutrition with n-3 PUFA supplementation gained on average 1.4 kg over the 2-week period versus a 0.1 kg weight loss in the standard EN group, but this difference was not statistically significant (*p* = 0.41). Early GCS recovery (day #7 GCS) was similar between study groups (6.0 vs. 5.8, *p* = 0.58). Both ICU and hospital LOS were decreased in the immunonutrition group, but this was not statistically significant. In-hospital mortality was lower in the immunonutrition group, but this was also not statistically significant (16.0% vs. 19.3%).

**Table 2.** Unadjusted Outcomes by Study Group

Outcome	Total (*n* = 151)	Immunonutrition EN group (*n* = 94)	Standard EN group (*n* = 57)	*p* value
Peak 14-day WBC	20.3 (6.6)	20.5 (6.5)	20 (6.9)	0.58
Peak 14-day CRP^a^	186 (90.9)	183 (92.0)	192 (90.8)	0.71
14-day Fever burden	15.1 (6.2)	15.1 (6.4)	15.2 (6.0)	0.75
Days with fever	8.5 (3.7)	8.4 (3.8)	8.6 (3.6)	0.74
Infection				
Any infection	82.8	80.9	86.0	0.42
Cerebrospinal fluid	0.7	1.1	0.0	0.43
Respiratory	78.2	77.7	79.0	0.85
Blood	17.2	13.8	22.8	0.16
Genitourinary	12.6	11.7	14.0	0.68
Wound	6.0	8.5	1.8	0.09
Ileus day #14	6.0	6.4	5.3	0.78
NPO days	3.8 (2.2)	3.7 (1.7)	4 (2.9)	0.59
Wt change (kg)	+0.8 (8.2)	+1.4 (8.2)	−0.1 (8.2)	0.41
GCS day #7	5.9 (2.5)	6 (2.5)	5.8 (2.4)	0.58
Inpatient mortality	17.2	16.0	19.3	0.6
Disposition				0.12
Acute care hospital	43.1	41.5	45.6	
SNF/assisted living	11.3	16.0	3.5	
Acute rehab	25.0	24.5	24.6	
Home	4.0	2.1	7.0	
Hospital LOS	38.4	25.1	43.8	0.3
ICU LOS	28.2	27.7	29.1	0.83

Results are displayed as means + SD for continuous variables and % for categorical variables.

^a^ CRP was analyzed in 63 patients with data available, 38 in the immunonutrition group, and 25 in the standard group.

WBC, white blood cell count, 10^9^/L; CRP, C-reactive protein, mg/L; NPO, nothing-by-mouth, no enteral nutrition given; Wt, weight; GCS, Glasgow Coma Scale score; SNF, skilled nursing facility; LOS length of stay; ICU, intensive care unit.

**FIG. 2.**
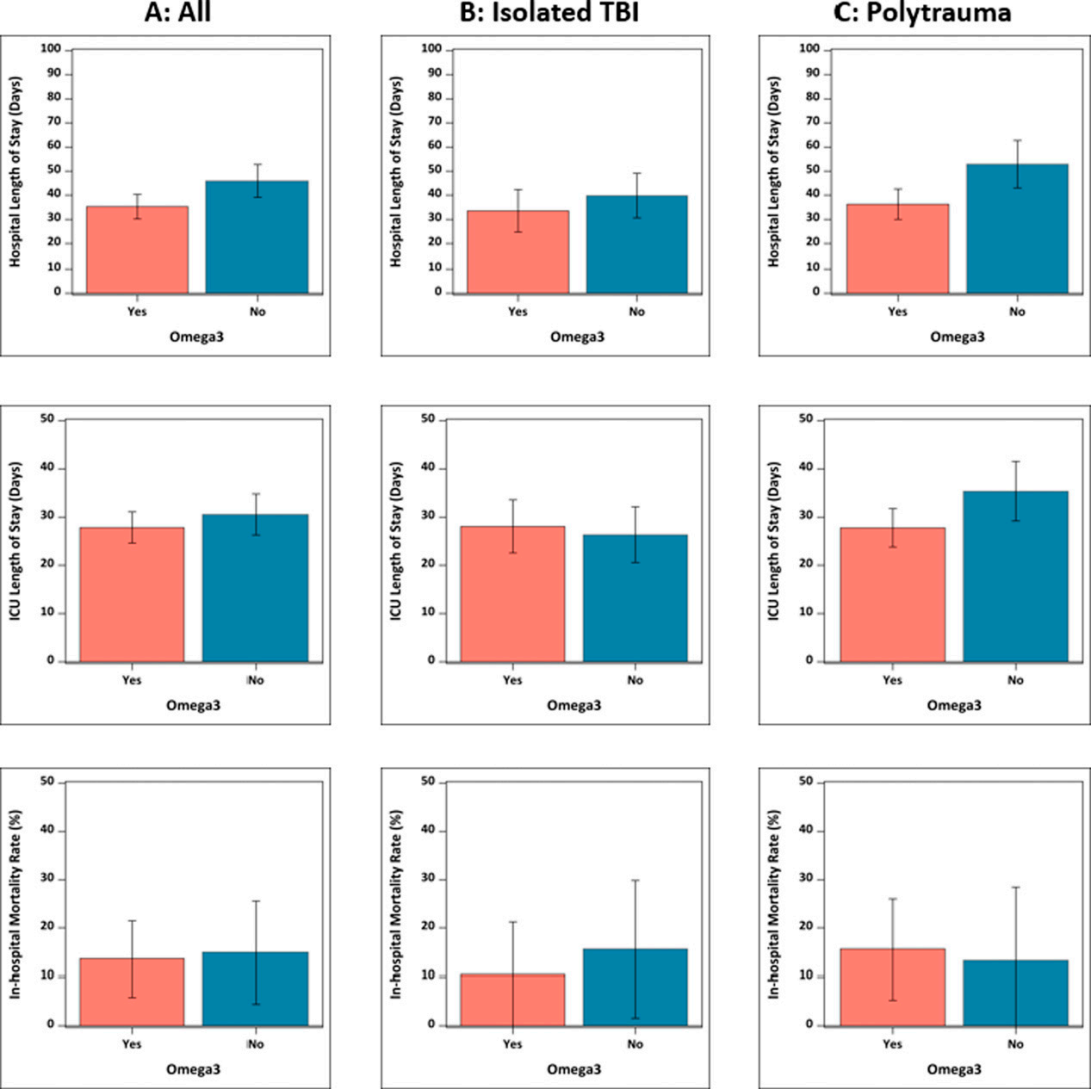
Adjusted Clinical Outcomes in the Whole Study Population and Subgroups. Results are displayed as bar charts with standard deviation bars for A: whole study population, B: isolated traumatic brain injury subgroup, C: polytrauma subgroup.

Clinical outcomes after adjusting for age, gender, and non-CNS emergency surgery are displayed in Table 3 and Figure 2. In multivariate linear regression analysis, hospital LOS was reduced in those prescribed immunonutrition with n-3 PUFA supplementation compared with standard EN (35.3 days versus 46.0 days, RR −10.7, 95% confidence interval [CI] −19.3, −2; *p* = 0.02). This difference was largest in the polytrauma subgroup (36.3 days vs. 52.9 days, RR −16.6, 95% CI −28.3, −4.9; *p* = 0.006). ICU LOS was not significantly different in the whole study population but was reduced in the polytrauma subgroup who received immunonutrition (27.8 days vs. 35.4 days, RR −7.6, 95% CI −7.6, 95% CI −15, −0.2; *p* = 0.04). In multivariate logistic regression, the risk of in-hospital mortality was similar between study groups. The RR of mortality was 0.7 in those prescribed immunonutrition compared with standard EN (10.4% vs. 15.7%), but this did not reach statistical significance (95% CI for RR −0.6, 1.9; *p* = 0.53).

**Table 3.** Multivariate Regression Analysis of Outcomes in Both the Whole Study Population and Subgroups

Outcome	Rate ratio	*p* value
Hospital LOS		
All	−10.7 (−19.3, −2.0)	0.02
Isolated TBI	−6.3 (−18.8, 6.2)	0.33
Polytrauma	−16.6 (−28.3, −4.9)	0.006
ICU LOS		
All	−2.7 (−8.1, 2.7)	0.33
Isolated TBI	1.7 (−6.2, 9.6)	0.67
Polytrauma	−7.6 (−15, −0.2)	0.04
In-hospital mortality		
All	0.9 (0, 1.8)	0.84
Isolated TBI	0.7 (−0.6, 1.9)	0.53
Polytrauma	1.2 (−0.2, 2.5)	0.81

Results are displayed as rate ratio (95% confidence interval).

LOS, length of stay; TBI, traumatic brain injury; ICU, intensive care unit.

## Discussion

In this retrospective descriptive cohort study of sTBI conducted at a single Level-I trauma center, those who received immunonutrition with n-3 PUFA-containing formulas for EN were slightly younger and more likely to be male, Hispanic/Latinx, have sustained non-CNS injury in addition to TBI, and have undergone emergent non-CNS surgery due to their injuries. The greater likelihood of being prescribed n-3 PUFA-containing immunonutrition in patients with polytrauma and in those requiring non-CNS surgery is not unexpected, given the high protein requirements believed to be necessary to support increased metabolic demand and promote wound healing after general traumatic injury and surgery.^8,14^ Further, the results also suggest that in clinical practice, the decision to prescribe EN with immunonutrition is not based on the presence of trauma alone. Specifically, older patients, female gender, and those with isolated TBI may not be considered to require similar nutritional interventions compared with younger male patients with polytrauma. Particularly for isolated TBI, this might be an incorrect assumption, as sTBI patients may need up to 200% of their baseline metabolic requirements,^15^ and with similarly high rates of malnourishment (68–76%) reported in both polytrauma and TBI populations.^8,16^ In addition, given inflammation and infectious risk are universal regardless of trauma type, further clinical study is needed to better define the nutritional needs and clinical targets for TBI patients, especially in those at high risk for malnutrition and poor functional outcomes.

Immunonutrition with n-3 PUFA supplementation compared with standard, polymeric EN formula was not associated with any statistically significant differences in clinical markers of inflammation and immunity. Specifically, peak 14-day WBC and CRP, 14-day fever burden and calendar days with fever were nearly identical between study groups. Although the incidence of culture-positive infection was lower in those receiving immunonutrition, this did not reach statistical significance (80.9% vs. 86.0%, *p* = 0.42). Given the proposed mechanism of immunonutrition with n-3 PUFA supplementation is to attenuate systemic inflammation and promote immune function, these results might be interpreted as unexpected; however, several circumstances should be considered. These outcome measures were chosen because they are assessable from retrospective medical record review but are likely nonspecific and crude measures of relevant anti-inflammatory activity that may be altered by several confounding factors. The study population was also severely injured (average GCS = 4.8), which may have blunted the effect of the nutritional intervention.

The culture-positive infection rate found in this study is higher than many hospital-acquired infection rates reported in moderate-to-severe TBI^17,18^ and hospitalized trauma patients.^19^ Both trauma and TBI are thought to increase the risk of in-hospital infection,^20,21^ while multiple surgical interventions further increase infection risk.^22^ Given the features of our study population, subjects were at high infection risk. Lastly, rates of infection in TBI populations are based on early retrospective report, whereas true infection risk is likely underestimated. Our reported rate of lower respiratory infection (78.2%) is comparable to recent literature described in a prospective surveillance study (72.2%).^23^ Given that nosocomial infection after TBI is associated with worse outcomes,^17,21^ the results also highlight the clinical problem of infection risk following TBI and the need to identify novel approaches to prevent this common complication.

Despite no identified differences in clinical markers of inflammation and immunity, there were important differences in clinical outcomes observed between the two study groups. In multivariate linear regression analysis, EN with immunonutrition and n-3 PUFA supplementation resulted in 10.7 fewer hospital days compared with standard EN (−19.3, −2.0, *p* = 0.02). In preplanned subgroup analysis, this appeared to be primarily driven by differences in those with polytrauma (−28.3, −4.9, *p* = 0.006). Similarly, ICU LOS was significantly shorter in the polytrauma subgroup. Although there was no statistically significant difference with in-hospital mortality between study groups in multivariate logistic regression analysis, there was a 0.7 rate ratio for mortality with treatment compared with standard nutrition (−0.6, 1.9, *p* = 0.53). From this study, it is unclear why immunonutrition with n-3 PUFA supplementation is associated with positive outcomes, but the results are sufficiently promising to warrant prospective study in clinical efficacy trials in this subgroup of the trauma population. It is possible that immunonutrition with n-3 PUFAs positively impacts energetics and optimizes nutritional status in ways that we were not possible to capture in a retrospective study. Particularly for n-3 PUFA supplementation, the pre-clinical data are robust. In rodent models of TBI, eicosanoid n-3 PUFA therapy has been demonstrated to be neuroprotective against inflammation-mediated oxidative stress and proapoptotic signaling after TBI,^24–26^ and can reduce proinflammatory signaling, maintain mitochondrial integrity, and reduce infarct size in stroke models.^27–29^ Additional amino acid components of immunonutrition, specifically arginine and glutamine, have been shown to support the synthesis of several cellular proteins vital to oxidative metabolism, immune function, and cellular repair.^30–32^

Although this was not a prospective study of early EN (<48–72 h from admission), prescription of enteral immunonutrition initiated with a mean of 50.8 h after admission in our study population appeared safe. Although a comprehensive evaluation of ICU complications was out of the scope of this study, the use of early EN immunonutrition therapy was not associated with weight loss, increased NPO days, day 14 ileus, or increased infection risk compared with standard therapy. This was an important finding as results from the general ICU population have been conflicting regarding the safety of early EN, partly due to a concern for aspiration-related respiratory infections.^33^ The capability to resolve inflammation without compromising immune function is one of the positive aspects of n-3 PUFA supplementation and immunonutrition, and because these components are naturally occurring with no known serious adverse effects, their supplementation is considered safe with a large therapeutic window.^12^ Early EN may be a clinically important intervention in TBI. It is estimated that every 10 kcal/kg/day increase in energy intake is associated with a 30–40% reduction in mortality risk.^34^

Important limitations to this study should be considered. In our retrospective analysis, data quality was dependent on the accuracy of electronic documentation. Given this anticipated limitation, study outcome measures were selected that were objective and were commonly documented as part of standard practice. This study lacked more precise measures of nutritional status, which could not be accurately measured by chart review. In addition, due to the retrospective nature of the study, we were unable to control all potential sources of bias and effect modification. The justification of selecting one nutrition intervention versus another was not standardized or documented. Nutrient components also differ between each EN formula, and we could not accurately control for % caloric, protein, fat, and carbohydrate needs met in retrospective analysis. Moreover, those who received EN with immunonutrition, including n-3 PUFA supplementation, may have received differential management compared with those receiving standard therapy based on the patient characteristics discussed. As with any observational study, correlation does not prove causation. Given the stringent inclusion criteria, the study population was relatively small. A larger study sample would provide a greater power in detecting clinically meaningful differences, especially in subgroups. Future blinded, randomized trials are needed to elucidate the direct impact of immunonutrition formulas on the inflammatory response and short- and long-term patient outcomes after moderate-to-severe TBI. Regarding generalizability, our US-based study population represented a very severe TBI cohort with a large proportion of Latinx and male subjects and may not be fully generalizable to other patient populations and regions of practice. The practicality of the intervention may also be influenced by the availability of specific EN products in different sites; however, n-3 supplementation products are widely available in many countries and are relatively low cost.

The emerging science in both pre-clinical and human populations of both n-3 PUFA supplementation and other amino acid immunonutrition warrants further study in prospective, pragmatic, randomized clinical efficacy trials. Additional correlations with biomarkers will be helpful in describing the molecular mechanisms as how immunonutrition improves clinical outcomes and to optimize proposed nutritional interventions. If mechanistic activity and positive impact on clinical outcomes can be demonstrated in clinical studies, routine incorporation of n-3 PUFA supplementation and immunonutrition with EN would be a novel approach to optimize ICU nutritional status and promote recovery after moderate or severe TBI in thousands of patients annually. Products that provide enriched amounts of n-3 PUFAs and amino acids are already in clinical practice, are widely available, and are relatively low cost, supporting their rapid translation and implementation into standards of ICU practice if benefit can be shown. Guideline recommendations do provide some support for these interventions in general ICU populations,^35,36^ but further rigorous study is needed in patients with TBI.^37^

## Conclusions

This study identifies patient characteristics of those who received immunonutrition EN with n-3 PUFA supplementation compared with standard, polymeric formulas. The use of immunonutrition was associated with important differences in hospital LOS and ICU LOS; however, the cause of this observed difference cannot be concluded from this study. Altogether, this suggests that there are potentially meaningful differences in patient characteristics and outcomes associated with the type of enteral feeding provided after sTBI. A prospective, pragmatic study of the intervention is warranted to confirm the biological mechanisms of treatment and elucidate their impact on patient outcomes. Our results directly inform the future design of such studies in TBI and other acute neurological disorders. Given the burden of disease and limited medical treatment options after moderate-to-severe TBI, novel approaches are urgently needed.

## Abbreviations Used

CAD: coronary artery disease
CI: confidence interval
CKD: chronic renal disease
CNS: central nervous system
CRP: C-reactive protein
CVA: cerebrovascular accident
DHA: docosahexaenoic acid
DM: diabetes mellitus
EDH: epidural hemorrhage
EMR: electronic medical record
EN: enteral nutrition
EPA: eicosapentaenoic acid
GCS: Glasgow Coma Scale
ICU: intensive care unit
IHT: isolated head trauma
IRB: institutional review board
LOS: length of stay
MVA: motor vehicle accident
n-3: omega-3
NPO: nothing by mouth
Ped: pedestrian
PUFA: polyunsaturated fatty acid
RR: relative risk
SAH: subarachnoid hemorrhage
SDH: subdural hemorrhage
SNF: skilled nursing facility
sTBI: severe traumatic brain injury
TBI: traumatic brain injury
US: United States
WBC: white blood cell count
Wt: weight
